# Supplementary material for: GEMsembler: consensus model assembly and structural comparison of genome-scale metabolic models across tools improve functional performance
Source: mSystems. 2025 Sep 8;10(10):e00574-25. doi: 10.1128/msystems.00574-25 (PMC12542698; doi:10.1128/msystems.00574-25)
Supplement: Supplemental material — Figures S1-S6, Table S7, supplemental model curation protocol, and list of supplemental tables. [file msystems.00574-25-s0001.pdf]

GEMsembler: consensus model assembly and structural comparison of genome-scale metabolic models across tools improve functional performance

Elena K. Matveishina[1,2], Bartosz J. Bartmanski[1], Sara Benito-Vaquerizo[1], Maria Zimmermann-Kogadeeva[1#]

[1] Genome Biology Unit, European Molecular Biology Laboratory (EMBL), Heidelberg, Germany.

[2] Collaboration for joint PhD degree between EMBL and Heidelberg University, Faculty of Biosciences, Heidelberg, Germany.

[#] Address correspondence to Maria Zimmermann-Kogadeeva: maria.zimmermann@embl.de

Supplementary materials

Contents:

- Model Curation Pipeline with GEMsembler
- Supplementary Figures S1-S6
- List of all Supplementary Tables and Files
- Supplementary Table 7

Model Curation Pipeline with GEMsembler

The following pipeline describes the main steps of genome-scale metabolic model curation with GEMsembler pipeline starting from the input models (e.g. models built with automatic tools, such as CarveMe, gapseq, or modelSEED from a genome sequence, or downloaded from the AGORA collection). In each step, we highlight whether it is performed automatically, or whether the user needs to make a decision based on GEMsembler pipeline outputs.

This pipeline is also provided under the following link:
https://grp-zimmermann-kogadeeva.embl-community.io/gemsembler/
Example notebook containing all the steps is provided at:
https://git.embl.org/grp-zimmermann-kogadeeva/GEMsembler/-/blob/master/docs/tutorial.ipynb

Model curation steps:

1. Collecting initial models (user-provided input)
2. Creating supermodel (automatic)
3. Assembling consensus models (automatic)
4. Initial check for production of all potential biomass components in known media (automatic, heatmap/table generated, user-provided media composition)
5. Biomass curation
    a. Table with model agreement on biomass components (automatic)
    b. Decisions on including each biomass component and adding the corresponding column with yes/no (manual, but based on automatically generated files from steps 5.a and 4 and optionally any other information)
    c. Use COBRApy functions to add/remove biomass components to implement the decision from the 5.b table (minimal coding)
6. Curation of growth in known media
    a. Checking for the production of final biomass components in all consensus models and corresponding pFBA pathways (automatic, summary heatmap/table and all pathways are generated in one function)
    b. Selecting a base consensus level (single decision based on step 6.a)
    c. Browsing through pathway maps (automatically generated at step 6.a) for each biomass component, not produced at the base consensus level and selecting reactions to add (manual)

   d. Combining base consensus with the selected reactions from step 6.c (automatic)
   e. Checking the production of each biomass component in the combined models with standard COBRApy functions for FBA analysis (minimal coding)
   f. Optionally selecting any other reactions to include/exclude in the final model based on the available knowledge.
   g. The final combination of base consensus and selected reactions (automatic)
   h. NGAM: setting ATPM reaction lower bound (optional, manual)
7. GPR curation with gene essentiality data (optionally, if data is available; automatically calculated in Python with supermodel: this functionality is not part of the GEMsembler package itself, but the code can be adapted from the code provided in the following repository https://git.embl.org/grp-zimmermann-kogadeeva/GEMsembler_paper/-/tree/master/Scripts scripts: ga_funcs.py, gen_alg.py, 05_ec_models_performance.py)
8. The output model(s) are saved in SBML format with the standard COBRApy command (optional, minimal coding).

Supplementary Figures

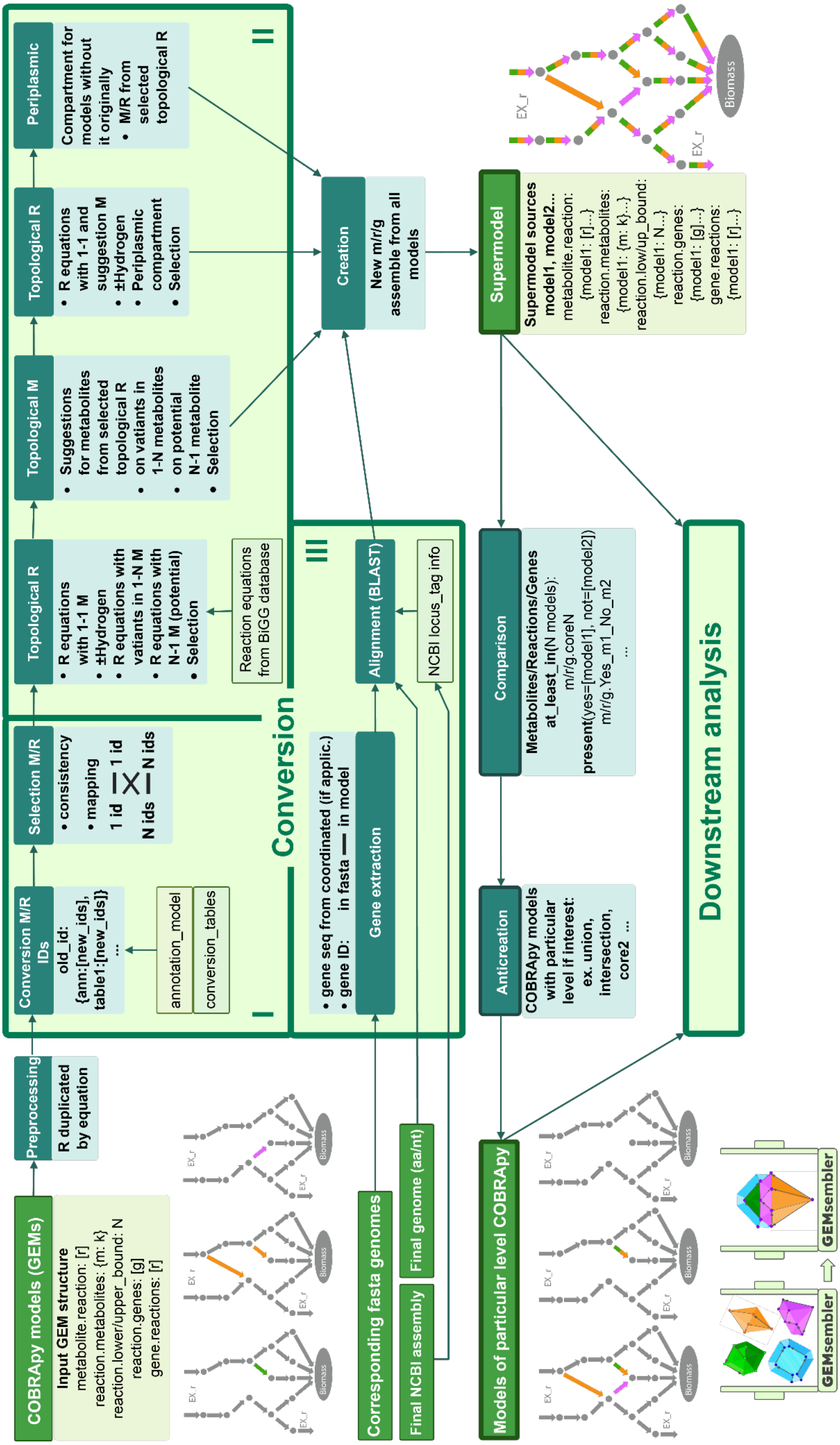

Figure S1. Schematic GEMsembler workflow.

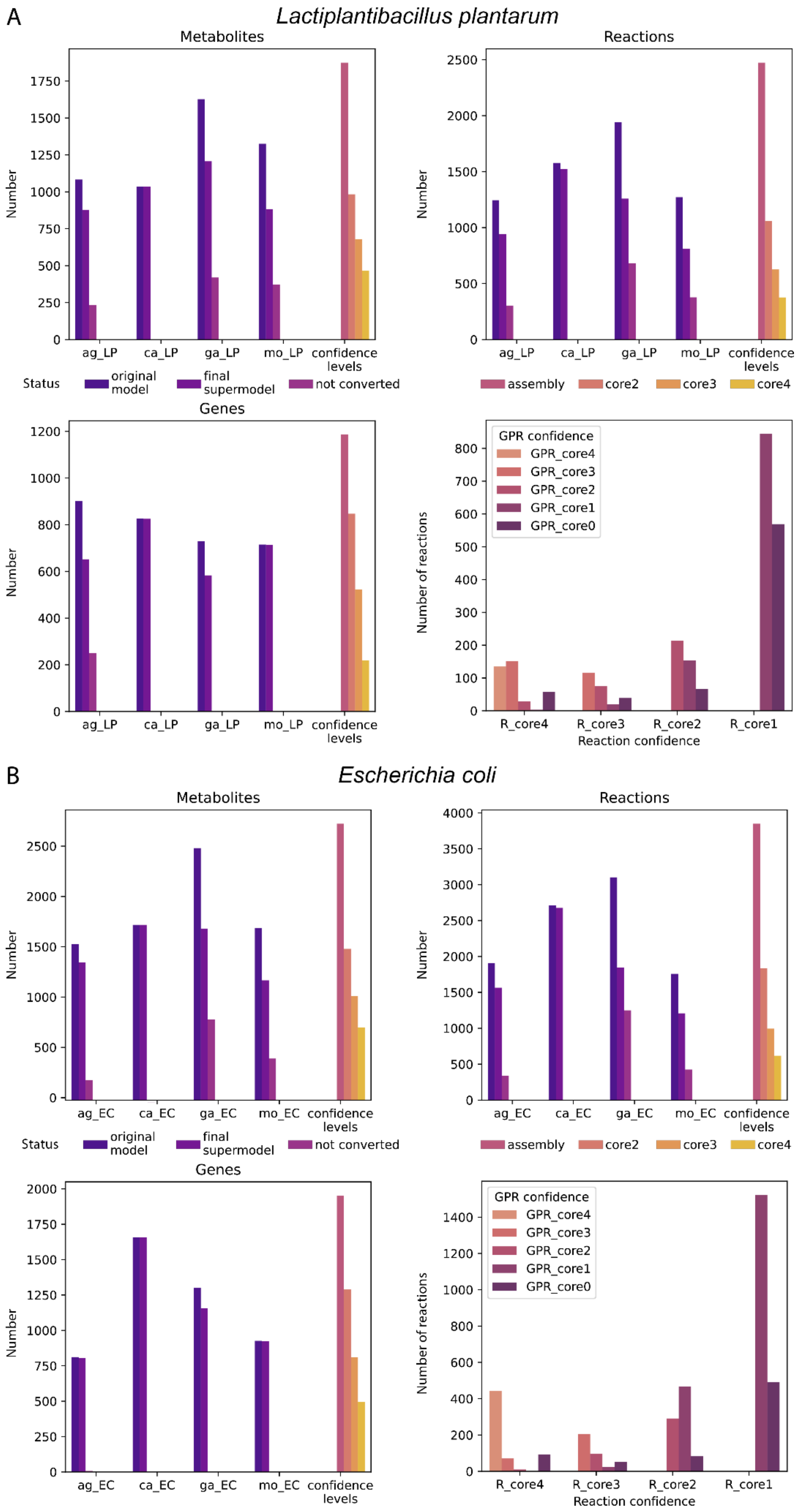

Figure S2. General characteristics of *L. plantarum* (A) and *E. coli* (B) models in terms of model agreement for metabolites, reactions, genes and GPRs. Ag: AGORA, ca: CarveMe, ga: gapseq, mo: modelSEED. CoreX corresponds to the agreement of X models.

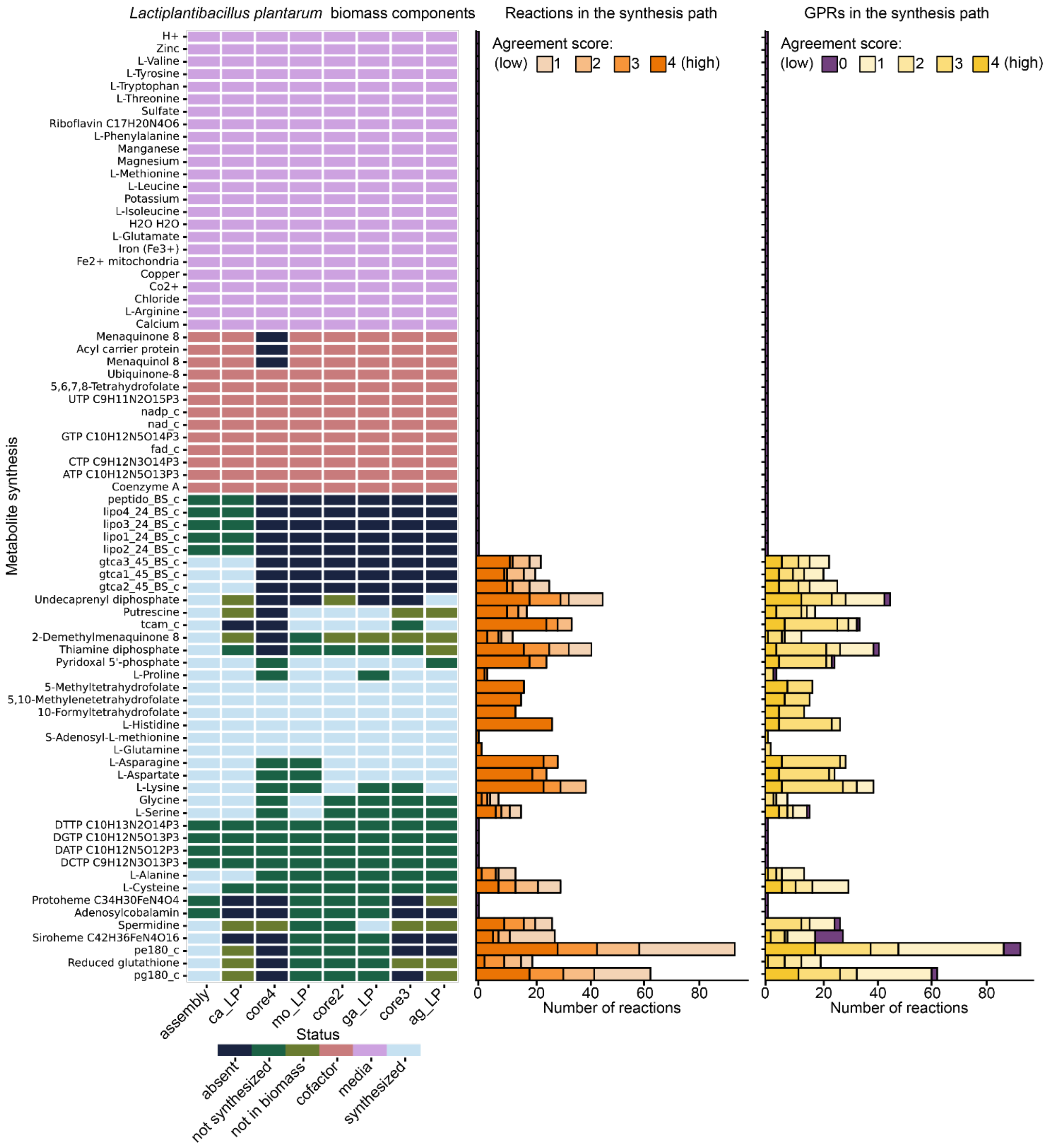

Figure S3. Biomass components production confidence for *L. plantarum* models. Ag: AGORA, ca: CarveMe, ga: gapseq, mo: modelSEED. CoreX corresponds to the agreement of X models.

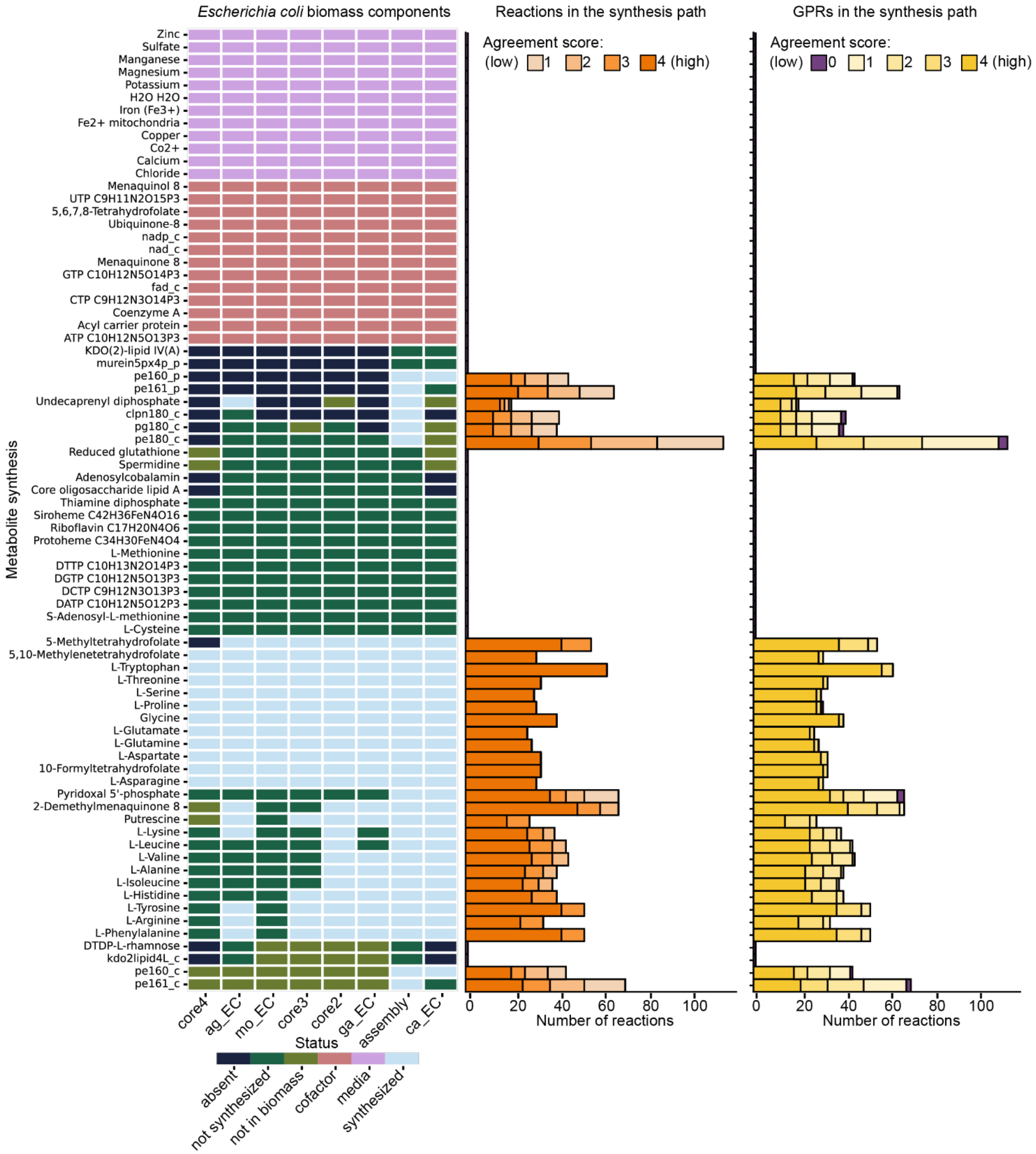

Figure S4. Biomass components production confidence for *E. coli* models. Ag: AGORA, ca: CarveMe, ga: gapseq, mo: modelSEED. CoreX corresponds to the agreement of X models.

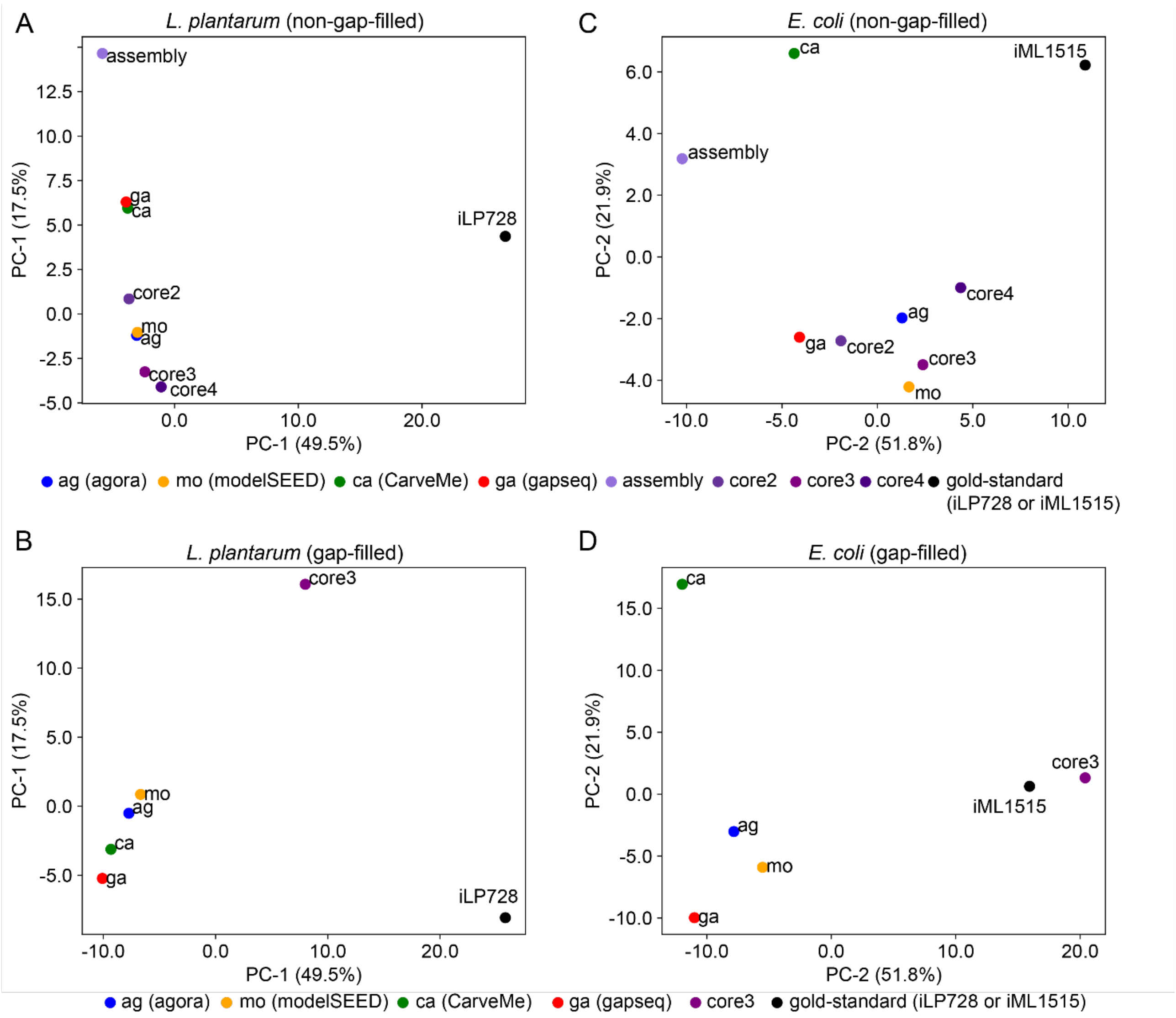

Figure S5. Principal component analysis of automatically reconstructed, consensus and gold-standard models based on reaction presence for *L. plantarum* (A, B) and *E. coli* (C, D). A, C – GEMsembler-converted models before gap-filling; consensus models before gap-filling, and original gold standard models. B, D – GEMsembler-converted original models gap-filled with CarveMe tool and curated core3 model (curation was performed to ensure growth in the corresponding minimal media of each bacterium). Only core3 model is depicted on B, D since only core3 was curated to ensure growth.

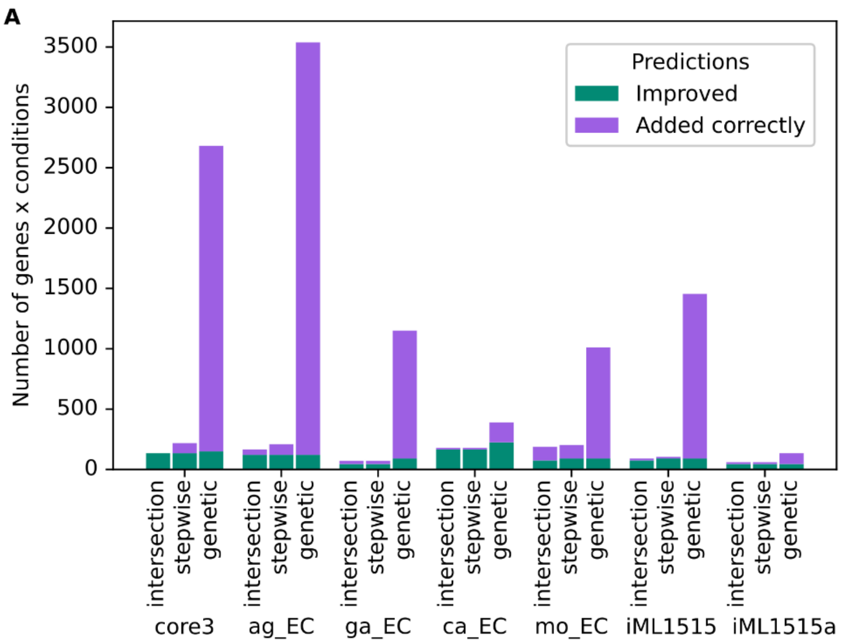

Figure S6. Improvement of gene essentiality prediction in *E. coli* with automatic GEMsembler GPR optimization procedure. Number of [genes x condition] pairs (for carbon sources) for which the essentiality prediction improved using the models modified with either SA or GA or both of them; and [genes x condition] pairs (for carbon sources) with correct essentiality predictions for which the genes were newly added to each model by these algorithms.

List of Supplementary tables

Abbreviations: EC - *Escherichia coli*, LP - *Lactiplantibacillus plantarum.*

1. Supplementary Table 1. General statistics on the numbers in categories from different metabolic models for
    a. Metabolites LP (figS2a)
    b. Reactions LP (figS2a)
    c. Genes LP (figS2a)
    d. Reaction-GPR pairs LP (figS2a)
    e. Metabolites EC (figS2b)
    f. Reactions EC (figS2b)
    g. Genes EC (figS2b)
    h. Reaction-GPR pairs EC (figS2b)
2. Supplementary Table 2. Topology-determined (MetQuest) production of central carbon metabolites and their confidence
    a. Production status table LP (fig2a).
    b. Production status table EC (fig2b)
    c. Path confidence LP (fig2a)
    d. Path confidence EC (fig2b)
    e. The most confident production LP
    f. The most confident production EC
    g. Production statistics LP
    h. Production statistics EC
3. Supplementary Table 3. Predefined central carbon metabolism pathways and their confidence
    a. Glycolysis LP
    b. Glycolysis EC
    c. Pentose phosphate pathway LP
    d. Pentose phosphate pathway EC
    e. Tricarboxylic acid cycle (TCA) LP
    f. Tricarboxylic acid cycle (TCA) EC
4. Supplementary Table 4. Topology-determined (MetQuest) production of biomass components and their confidence
    a. Production status table LP (figS3a)
    b. Production status table EC (figS3b)
    c. Path confidence LP (figS3a)
    d. Path confidence EC (figS3b)
    e. The most confident production LP
    f. The most confident production EC
    g. Production statistics LP
    h. Production statistics EC
5. Supplementary Table 5. Biomass reaction composition, its confidence and decision on inclusion in the final biomass reaction
    a. Biomass composition and decision LP
    b. Biomass composition and decision EC

6. Supplementary Table 6. Summary of topology-based confidence analysis and identification of uncertainties
    a. Classification for metabolites of interest and their biosynthesis (fig2C)
    b. The most unconfident reactions (fig2D)
    c. Example of succinate biosynthesis LP (fig2E)
    d. Example of valine biosynthesis EC (fig2F)
7. Supplementary Table 7. Media composition (inside this file)
    a. PMM5 minimal media LP; CDPM media LP; M9 minimal media EC
8. Supplementary Table 8. Flux-determined (FBA/pFBA) production of biomass components and their confidence
    a. Production status table final biomass LP (fig3a)
    b. Production status table final biomass EC (fig3b)
    c. Example of interactive map for thiamine diphosphate curation (fig 3c)
    d. Production status table LP
    e. Production status table EC
    f. Production status table mixed LP (with non-converted features from the original model added to the converted model)
    g. Production status table mixed EC (with non-converted features from the original model added to the converted model)
9. Supplementary Table 9. LP models performance and comparison with gold-standard model
    a. Similarity table for reactions and the corresponding genes (fig4a)
    b. Number of reactions and genes from different models (fig4a)
    c. Auxotrophy growth status (fig4b) (0 - no growth, 1 - reduced growth, 2 - growth)
    d. Auxotrophy growth FBA simulations (flux value through the biomass reaction)
    e. Auxotrophy models performance (1 - match with the experimental data; 0 - mismatch)
10. Supplementary Table 10. EC models performance and comparison with gold-standard models
    a. Similarity table for reactions and the corresponding genes (fig5a)
    b. Number of reactions and genes from different models (fig5a)
    c. Models growth on different carbon sources
    d. AUCPR for each step in SA (stepwise procedure algorithm)
    e. Number of generations intersected for GA (genetic algorithm) solution
    f. Number of GPR changed by SA or GA (fig5d)
    g. Number of genes changed by SA or GA (fig5e)
    h. Status of gene essentiality predictions (fig5f)
    i. Gene prediction improvement (fig5g)
    j. Gene-condition pairs prediction improvement (figS4)
    k. Models growth on different nitrogen sources

All production tables (in Tables S2, S4, S8) use the following numeric code for production status:
5 - synthesised; 4 - in the media; 3 - cofactor; 2 - not in the biomass; 1- not synthesised; 0 - not in the model.

List of Supplementary Files

Supplementary files contain interactive pathway maps generated by GEMsembler in HTML format that were used to produce the results and are referred to in the text. The files are available on Zenodo: https://doi.org/10.5281/zenodo.16529342 and on GitLab at https://git.embl.org/grp-zimmermann-kogadeeva/GEMsembler_paper/-/tree/master/Output/Supplementary_files.

Abbreviations: EC - *Escherichia coli*, LP - *Lactiplantibacillus plantarum.*

Supplementary Files list:

1. Glycolysis LP
2. Glycolysis EC
3. Pentose phosphate pathway LP
4. Pentose phosphate pathway EC
5. Tricarboxylic acid cycle (TCA) LP
6. TCA EC
7. Succinate biosynthesis LP (Fig. 2E)
8. Valine biosynthesis EC (Fig. 2F)
9. Thiamine diphosphate pFBA pathway LP (Fig. 3C)
10. Pyridoxal 5'-phosphate biosynthesis with pyridoxamine from published iLP728 _LP model
11. Pyridoxal 5'-phosphate biosynthesis without pyridoxamine from GEMsembler-curated core3 LP model
12. Glutamate biosynthesis with pyridoxamine from published iLP728 LP model

Supplementary Table 7. Media compositions (in CarveMe format). This table is also available as a text file on Zenodo: https://doi.org/10.5281/zenodo.16529342 and on GitLab at https://git.embl.org/grp-zimmermann-kogadeeva/GEMsembler_paper/-/tree/master/Output/Supplementary_tables.

**medium**	**description**	**compound**	**name**
CDPM	pi	pi	pi
CDPM	glc__D	glc__D	glc__D
CDPM	na1	na1	na1
CDPM	ac	ac	ac
CDPM	nh4	nh4	nh4
CDPM	cit	cit	cit
CDPM	ascb__L	ascb__L	ascb__L
CDPM	ala__L	ala__L	ala__L
CDPM	arg__L	arg__L	arg__L
CDPM	asp__L	asp__L	asp__L
CDPM	cys__L	cys__L	cys__L
CDPM	glu__L	glu__L	glu__L
CDPM	gly	gly	gly
CDPM	his__L	his__L	his__L
CDPM	ile__L	ile__L	ile__L
CDPM	leu__L	leu__L	leu__L
CDPM	lys__L	lys__L	lys__L
CDPM	met__L	met__L	met__L
CDPM	phe__L	phe__L	phe__L
CDPM	pro__L	pro__L	pro__L
CDPM	ser__L	ser__L	ser__L
CDPM	thr__L	thr__L	thr__L
CDPM	trp__L	trp__L	trp__L
CDPM	tyr__L	tyr__L	tyr__L
CDPM	val__L	val__L	val__L
CDPM	lipoate	lipoate	lipoate
CDPM	btn	btn	btn
CDPM	nac	nac	nac

CDPM	pnto__R	pnto__R	pnto__R
CDPM	4abz	4abz	4abz
CDPM	pydam	pydam	pydam
CDPM	pydxn	pydxn	pydxn
CDPM	ribflv	ribflv	ribflv
CDPM	thm	thm	thm
CDPM	adocbl	adocbl	adocbl
CDPM	ade	ade	ade
CDPM	gua	gua	gua
CDPM	ins	ins	ins
CDPM	xan	xan	xan
CDPM	orot	orot	orot
CDPM	thymd	thymd	thymd
CDPM	ura	ura	ura
CDPM	mg2	mg2	mg2
CDPM	cl	cl	cl
CDPM	ca2	ca2	ca2
CDPM	mn2	mn2	mn2
CDPM	fe3	fe3	fe3
CDPM	fe2	fe2	fe2
CDPM	zn2	zn2	zn2
CDPM	so4	so4	so4
CDPM	cobalt2	cobalt2	cobalt2
CDPM	cu	cu	cu
CDPM	mobd	mobd	mobd
CDPM	k	k	k
CDPM	cu2	cu2	cu2
CDPM	h2o	h2o	h2o
CDPM	h	h	h
PMM5	pi	pi	pi
PMM5	glc__D	glc__D	glc__D
PMM5	na1	na1	na1
PMM5	ac	ac	ac

PMM5	ascb__L	ascb__L	ascb__L
PMM5	arg__L	arg__L	arg__L
PMM5	glu__L	glu__L	glu__L
PMM5	ile__L	ile__L	ile__L
PMM5	leu__L	leu__L	leu__L
PMM5	met__L	met__L	met__L
PMM5	phe__L	phe__L	phe__L
PMM5	thr__L	thr__L	thr__L
PMM5	trp__L	trp__L	trp__L
PMM5	tyr__L	tyr__L	tyr__L
PMM5	val__L	val__L	val__L
PMM5	nac	nac	nac
PMM5	pnto__R	pnto__R	pnto__R
PMM5	ribflv	ribflv	ribflv
PMM5	mg2	mg2	mg2
PMM5	cl	cl	cl
PMM5	ca2	ca2	ca2
PMM5	mn2	mn2	mn2
PMM5	fe3	fe3	fe3
PMM5	fe2	fe2	fe2
PMM5	zn2	zn2	zn2
PMM5	so4	so4	so4
PMM5	cobalt2	cobalt2	cobalt2
PMM5	cu	cu	cu
PMM5	mobd	mobd	mobd
PMM5	k	k	k
PMM5	cu2	cu2	cu2
PMM5	h2o	h2o	h2o
PMM5	h	h	h
M9	glc__D	glc__D	glc__D
M9	pi	pi	pi
M9	co2	co2	co2
M9	mg2	mg2	mg2

M9	cl	cl	cl
M9	ca2	ca2	ca2
M9	mn2	mn2	mn2
M9	fe3	fe3	fe3
M9	fe2	fe2	fe2
M9	zn2	zn2	zn2
M9	so4	so4	so4
M9	cobalt2	cobalt2	cobalt2
M9	mobd	mobd	mobd
M9	k	k	k
M9	cu2	cu2	cu2
M9	h2o	h2o	h2o
M9	h	h	h
M9	ni2	ni2	ni2
M9	sel	sel	sel
M9	nh4	nh4	nh4
M9	na1	na1	na1
M9	o2	o2	o2
M9	tungs	tungs	tungs
M9	slnt	slnt	slnt
